# Genetic and Pathogenic Characterization of a Porcine Deltacoronavirus Strain Isolated in Zhejiang Province, China

**DOI:** 10.1155/tbed/4084814

**Published:** 2025-05-18

**Authors:** Ya-Qing Zhang, Bin Wang, Weiqun Wei, Wan Lu, En-Zhong Du, Yan Liu, Yong-Le Yang, Yao-Wei Huang

**Affiliations:** ^1^Department of Veterinary Medicine, Zhejiang University, Hangzhou 310058, China; ^2^Xianghu Laboratory, Biomanufacturing Institute, Hangzhou 311231, China; ^3^Guangdong Laboratory for Lingnan Modern Agriculture, College of Veterinary Medicine, South China Agricultural University, Guangzhou 510642, China; ^4^Jiangxi Tianjia Bioengineering Co., Ltd., Nanchang 330200, China; ^5^YEBIO Bioengineering Co., Ltd. of Qingdao, Qingdao 266114, China

**Keywords:** cross-species transmission, pathogenicity, phylogenetics, porcine deltacoronavirus

## Abstract

Porcine deltacoronavirus (PDCoV) has emerged as a significant pathogen in swine, affecting animal health and posing potential risks for cross-species transmission. In this study, we successfully isolated a PDCoV strain named HZYH-2019 from the feces of diarrheal sows in Zhejiang Province, China. The viral growth curve demonstrated strong adaptation of this strain to cells, with particularly high replication efficiency observed in LLC-PK1 cells. Genomic analysis revealed a high degree of nucleotide sequence similarity between PDCoV HZYH-2019 and other PDCoV strains. A notable mutation at the tenth amino acid position of the spike protein altered the predicted signal peptide position. Phylogenetic analyses indicated that PDCoV HZYH-2019 clustered with Chinese strains, while four Chinese strains were grouped within the American spectrum, suggesting that the pork trade may facilitate cross-border virus transmission. Analysis of known PDCoV strains inferred that PDCoV may have originated in Asia and that there is cross-species transmission from birds to mammals. Notably, PDCoV HZYH-2019 caused diarrhea in piglets without mortality, although significant intestinal lesions were observed. These findings enhance our understanding of PDCoV's biological behavior and zoonotic potential, informing the development of effective vaccines and control measures to manage future outbreaks.

## 1. Introduction

Porcine deltacoronavirus (PDCoV) is an emerging pathogen belonging to the family *Coronaviridae*, genus *deltacoronavirus* [1]. It is an enveloped, single-stranded, positive-sense RNA virus that has garnered significant attention due to its impact on the swine industry and potential for cross-species transmission. The genome structure of PDCoV is arranged as follows: 5′-untranslated region (UTR), open reading frames 1a and 1b (ORF1a/1b), spike protein (S), envelope protein (E), membrane protein (M), nonstructural protein 6 (NS6) [2], nucleocapsid protein (N), NS7, NS7a, and the 3′-UTR. PDCoV was first identified in Hong Kong in 2012 without any associated clinical symptoms [3]. It was not isolated until 2014, when it was first isolated in the feces of diarrheic pigs in Ohio, USA [4–6]. Since then, PDCoV has been detected in multiple countries, leading to outbreaks characterized by diarrhea and vomiting in piglets, resulting in significant economic losses [7–11].

To date, all other members of the genus *deltacoronavirus* have been detected in birds, suggesting that birds are the natural and ancestral hosts of deltacoronavirus [12]. Although PDCoV was initially detected in pigs, recent studies have indicated that it may have an avian origin, arising from recombination between HKU17 and bulbul coronavirus HKU11 [3, 13, 14]. In fact, PDCoV has demonstrated a broad species tropism in both cell and animal experiments. It can infect calves [15], mice [16], chickens [17], and turkeys [18], and it is capable of growing in various cell types derived from pigs, humans, and birds [16]. Notably, PDCoV was first detected in the plasma of children with acute febrile illness in Haiti [19], indicating its potential to cross species barriers and possibly trigger a new pandemic.

Understanding the genomic characterization and evolutionary dynamics of PDCoV is critical for elucidating its transmission patterns and developing effective control measures. Previous research has highlighted the virus's propensity for genetic recombination and mutation, which may facilitate host-switching events and adaptation to new environments [20–22]. While the specific mechanisms underlying these processes are complex and remain an area for future research, this study focuses on analyzing the genomic features and evolutionary trajectory of PDCoV to better understand its potential for cross-species transmission.

In this study, we investigate the PDCoV strain HZYH-2019, isolated from pigs, to elucidate its genomic features, evolutionary trajectory, and host interactions. Our phylogenetic analysis, focusing on the S gene, provides a detailed view of the virus's evolutionary rate and geographic origin. A key aspect of our research is exploring recombination and host-switching events, particularly concerning avian species, to assess the risk of cross-species transmission and the emergence of new variants. Additionally, we examine codon usage bias in deltacoronaviruses to infer evolutionary pressures and host adaptation strategies. We also evaluate its pathogenicity in neonatal piglets. These findings are crucial for developing effective prevention and control strategies, enhancing our understanding of PDCoV's biology and epidemiology, and contributing to broader efforts in managing this emerging viral threat.

## 2. Materials and Methods

### 2.1. Clinical Sample Collection and Treatment

At the end of 2019, fecal samples were collected from sows with diarrhea at a farm in Yuhang District, Hangzhou, and tested for intestinal viruses using RT-PCR. Samples were then performed following established protocols with modifications [23]. Briefly, PDCoV-positive fecal samples were homogenized in Dulbecco's Modified Eagle Medium (DMEM; Gibco) supplemented with 1× penicillin–streptomycin (10,000 U/mL). The homogenate was subjected to centrifugation to remove large particulates at 4°C. The supernatant was filtered through a 0.22 μm filter.

### 2.2. Virus Isolation

PDCoV was isolated using the LLC-PK1 cell line (ATCC CLR-101). LLC-PK1 cells were grown in DMEM with 10% fetal bovine serum and 1% antibiotics (penicillin, streptomycin). For PDCoV proliferation, the maintenance medium was DMEM with 1% antibiotics and 5 µg/mL trypsin.

When the LLC-PK1 cell monolayer in a six-well plate reached 80%–90% confluence, it was washed three times with PBS. 500 µL of filtered supernatant and 1.5 mL of maintenance medium were added to each well. After virus adsorption at 37°C for 1 h, the cells were washed three times with PBS, and 2 mL of maintenance medium was added to each well. The cells were incubated at 37°C under 5% CO_2_. When 80%–90% of the cells exhibited significant cytopathic effects (CPE), including aggregation, rounding, and crumpling, typically observed at 1–2 days postinoculation (dpi), the plates were subjected to two freeze–thaw cycles at −80°C. The supernatant was stored at −80°C as seed stock for future passaging.

### 2.3. Immunofluorescence Assay

LLC-PK1 cells in 24-well culture plates were infected with PDCoV at a multiplicity of infection (MOI) of 0.1. 24 h postinoculation (hpi), the cells were fixed with 4% paraformaldehyde for 15 min and then permeabilised with 0.1% Triton X-100 for 10 min at room temperature. The cells were incubated with a monoclonal antibody specific to the PDCoV-N protein for 1 h. This was followed by incubation with a 1:1000 dilution of Alexa Fluor 488-conjugated goat antimouse IgG in 1x PBS for 1 h at 37°C, shielded from light. Finally, nuclei were stained with DAPI for 5 min at room temperature. After washing with PBS, the stained cells were observed under a fluorescence microscope.

### 2.4. Viral Replication Kinetics in LLC-PK1 Cells and ST Cells

LLC-PK1 cells at 90% confluence in 24-well plates were inoculated with PDCoV at an MOI of 0.1. After adsorption for 1 h in a 37°C CO_2_ incubator, the cells were washed three times with PBS, and then 0.5 mL of maintenance medium was added to each well. At 2, 12, 24, 36, 48, and 60 hpi, supernatants were harvested and PDCoV titers were determined by 50% tissue culture infective dose (TCID_50_) [24].

### 2.5. RNA Isolation and Reverse Transcriptase Quantitative PCR (RT-qPCR)

Total RNA was isolated from cells or tissues using Trizol (Thermo Fisher Scientific, USA) according to the manufacturer's instructions. The RNA titer of PDCoV was assessed using RT-qPCR targeting the membrane (M) gene. Specific primers were employed, with the following sequences: 5′-ATCGACCACATGGCTCCAA-3′ and 5′-CAGCTCTTGCCCATGTAGCTT-3′. Additionally, a FAM-labeled probe, CACACCAGTCGTTAAGCATGGCAAGCT-BHQ, was used. The RT-qPCR protocol used was based on previously published methods [24, 25].

### 2.6. Electron Microscopy

Supernatant from PDCoV-infected cell cultures exhibiting CPEs was purified and subjected to negative staining. The specimens were adsorbed onto carbon-coated copper grids, treated with 2% sodium phosphotungstic acid for 1.5 min, and subsequently examined using a Hitachi H-7650 transmission electron microscope.

### 2.7. PDCoV Sequence Alignment and Maximum Likelihood (ML) Phylogenetic Tree Construction

Genome sequences for PDCoV and avian deltacoronavirus (ADCoV) were obtained from NCBI GenBank (https://www.ncbi.nlm.nih.gov/), along with a PDCoV isolate from Zhejiang, sequenced in our laboratory (designated PDCoV HZYH-2019, GenBank No. PQ645844). A total of 143 PDCoV reference sequences (Table S1) and 27 ADCoV strains (Table S2) were compiled. Multiple sequence alignments of the viral whole genome or partial genes were performed using the MAFFT program in BioAider v1.314 software [26]. ML phylogenetic trees were constructed using IQ-tree v1.6.12 with a bootstrap value of 1000. The optimal nucleotide substitution model was automatically determined by the program based on the Bayesian Information Criterion (BIC) [27]. Analysis results were visualized using the iTOL website (https://itol.embl.de/) [28].

### 2.8. PDCoV Differentiation Time Estimation

Initially, viral sequences were assessed for sufficient temporal signal using TempEst v1.5.3 [29]. Subsequently, aligned nucleotide sequences were imported into ModelFinder within PhyloSuite v1.2.2 to determine the optimal substitution model for BEAST based on the BIC. The optimal model was selected by comparing combinations of molecular clock and tree prior models using marginal likelihood ratios estimated through path sampling.

In BEAST v1.10.4, Markov Chain Monte Carlo (MCMC) methods were used to estimate the Time to the Most Recent Common Ancestor (tMRCA) and the evolutionary rate. Based on existing studies on the evolutionary rate of PDCoV S genes [20], a uniform prior for the evolutionary rate was set between 1 × 10^−4^ and 1 × 10^−2^ nucleotide substitutions per site per year. The sampling frequency was set to 50,000, with a chain length of 500,000,000, discarding the first 10% of samples to mitigate burn-in effects.

The effective sample size (ESS) of all estimated parameters was evaluated using Tracer v1.6 to ensure ESS values exceeded 200 for convergence [30]. Finally, in TreeAnnotator v1.10.4, the top 10% of the Bayesian maximum clade credibility (MCC) tree was discarded, and the final MCC tree was visualized using FigTree v1.4.3.

### 2.9. Recombination Analysis

Seven different scanning methods in RDP4 v4.1.0.1 software, including RDP, GENECONV, BootScan, MaxChi, Chimaera, SIScan, and 3Seq, were used to detect recombination events. A *p*-value threshold of less than 0.05 was set, requiring at least three assays to be positive to confirm a true recombination event [20, 31]. Identified recombination events were further confirmed using Simplot v3.5.1.

### 2.10. Analysis of Virus-Host Coevolution

Phylogenetic trees for host species (porcine and avian) were constructed using the TIMETREE website (http://www.timetree.org/). The host evolutionary tree was imported into JANE software alongside the virus evolutionary tree to construct the host-virus mapping relationship [32]. The host-virus relationship was categorized into five events: cospeciation, duplication, duplication with host switch, loss, and failure to diverge. Cost values were set for each event, with default values of 0, 1, 2, 1, and 1, respectively.

### 2.11. Codon Usage Analysis

The average guanine and cytosine (G + C) content, along with the GC content at the first and second positions of synonymous codons (GC12s) and the third position (GC3s), are key indicators of codon usage bias. Viral codon usage patterns are often closely related to host genomic characteristics, making correlation analyses useful for assessing potential viral cross-species transmission [33, 34]. Coding sequences of selected viral genomes were analyzed using CodonW 1.4.2 (codonw.sourceforge.net/) to calculate GC content, GC12s, GC3s, effective codon count (ENC), and relative synonymous codon usage (RSCU).

Scatter plots with GC3 as the *x*-axis and GC12 as the *y*-axis were generated using GraphPad Prism 8, with correlation curves fitted to analyze their relationship. Another scatter plot was created with GC3s as the *x*-axis and ENc as the *y*-axis, and a standard curve was plotted using the formula ENc = 2 + GC3s + 29/(GC3s^2^ + (1 − GC3s)^2^) [35]. If scatter points lie on the standard curve, it indicates that base composition determines codon preference, not natural selection. RSCU heatmaps were generated using TBtools v1.098689 to compare RSCU differences among different deltacoronaviruses.

### 2.12. Animal Experiments

All animal experiments were conducted with the approval of the Institutional Animal Care and Use Committee (approval number 2022ZAASLA87). Twelve 5-day-old piglets were purchased from Yunmu Farm (Jiangsu, China). Fecal samples from these piglets tested negative for PDCoV, porcine epidemic diarrhea virus (PEDV), transmissible gastroenteritis virus (TGEV), and swine acute diarrhea syndrome coronavirus (SADS-CoV) RNA as described previously [23, 36]. Sera from sows and piglets tested negative for neutralizing antibodies against PDCoV. Twelve 5-day-old piglets were randomly allocated into two groups. One group (*n* = 6) was orally inoculated with PDCoV at a dose of 10^6^ TCID_50_/mL, with 3 mL administered per piglet. The other group (*n* = 6) received an oral administration of DMEM and served as the mock infection group. Piglets were observed daily for clinical signs, including diarrhea and vomiting episodes. To accurately quantify the tissue-specific viral load, groups of three piglets were humanely euthanized at 3 and 5 dpi. Euthanasia was performed by CO_2_ asphyxiation in a controlled chamber, with exposure duration confirmed to ensure unconsciousness prior to death. Subsequently, a systematic necropsy was performed, and intestinal tissues (duodenum, jejunum, and ileum) and lymphoid tissues (mesenteric lymph nodes) were collected. Each tissue sample was dissected, weighed, and homogenized in medium using 1.0 mm zirconia/silica beads.

### 2.13. Histopathology and Immunohistochemistry (IHC)

Ileal tissue samples were fixed in 4% paraformaldehyde, followed by dehydration, paraffin embedding, sectioning, and mounting onto slides. Sections were stained with hematoxylin and eosin (HE), and observations were made using an inverted fluorescence microscope. IHC was performed following standard laboratory protocols [37]. To detect PDCoV-specific antigens, selected paraffin-embedded sections were treated with a PDCoV-N-specific monoclonal antibody (dilution 1:500) and a secondary antibody, HRP-conjugated goat antimouse IgG (dilution 1:1000).

### 2.14. Statistical Analysis

Results are expressed as the mean ± standard deviation unless otherwise indicated. Statistical analyses were conducted using Student's *t*-test with GraphPad Prism 8 software (GraphPad Software, San Diego, CA, USA). A *p*-value of less than 0.05 was considered statistically significant for group differences.

## 3. Results

### 3.1. Isolation, Characterization, and Growth Kinetics of PDCoV Strain HZYH-2019 in LLC-PK1 and ST Cells

**Figure 1:**
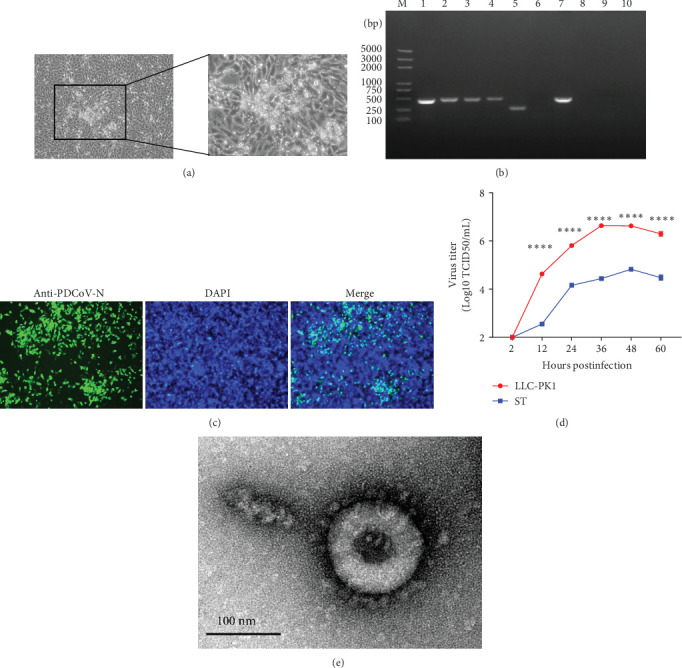
Isolation and characterization of PDCoV. (a) Cytopathic effect (CPE) observed in LLC-PK1 cells infected with PDCoV HZYH-2019. (b) RT-PCR for virus identification in fecal samples. Lane 1: PEDV positive control; lane 2: PDCoV positive control; lane 3: SADS-CoV positive control; lane 4: TGEV positive control; lane 5: PToV positive control; lane 6: PEDV; lane 7: PDCoV; lane 8: SADS-CoV; lane 9: TGEV; and lane 10: PToV. (c) IFA staining of PDCoV HZYH-2019-inoculated LLC-PK cells (scale bar: 100 μm). (d) Growth curves of LLC-PK1 and ST cells infected with PDCoV HZYH-2019 at an MOI of 0.1. The data are presented as the mean ± standard deviation. *p*-values were determined using an unpaired two-tailed Student's *t*-test. The significance level was set at *⁣*^*∗*^*⁣*^*∗*^*⁣*^*∗*^*⁣*^*∗*^: *p* ≤ 0.0001. (e) Electron microscope image of the PDCoV strain HZYH-2019 using phosphotungstic acid negative staining. Bar = 100 nm.

In late 2019, a severe diarrhea outbreak occurred at a pig farm in Xianju County, Zhejiang Province, resulting in the death of all affected piglets. A strain of PDCoV was successfully isolated from fecal samples of sows exhibiting diarrhea, and whole-genome sequencing was conducted, designating the strain as HZYH-2019. Initially, LLC-PK1 cells inoculated with fecal samples containing HZYH-2019 exhibited a distinct CPE within 24 h, characterized by cell enlargement and rounding (Figure 1a). The isolate was subjected to plaque purification at the third passage. RT-PCR analysis confirmed the presence of PDCoV, whereas tests for other porcine enteric viruses, including PEDV [36], SADS-CoV [23, 38], TGEV, and porcine toroviruses (PToV) [39], yielded negative results (Figure 1b). Infection of LLC-PK1 cells with the P3 passage of the PDCoV strain HZYH-2019 was further validated through IFA using a monoclonal antibody targeting the PDCoV N protein, with specific immunofluorescence detected in most cells 24 hpi (Figure 1c). To further elucidate the growth kinetics of the PDCoV HZYH-2019 P3 strain in LLC-PK1 and ST cells, infections were conducted at a MOI of 0.1. Supernatants were collected at various time points: 2, 12, 24, 36, 48, and 60 hpi. A significant increase in viral titer was observed in both cell lines at 12 hpi. In LLC-PK1 cells, viral titers peaked at 36 hpi and subsequently stabilized. Conversely, in ST cells, viral titers reached its peak at 48 hpi and then stabilized (Figure 1d). Notably, PDCoV HZYH-2019 proliferation was significantly greater in LLC-PK1 cells than in ST cells, indicating a cell-type-specific growth advantage. Electron microscopy analysis of PDCoV HZYH-2019 virions purified from the supernatant of infected LLC-PK1 cell cultures revealed characteristic coronavirus morphology, with particles measuring 120–130 nm in diameter (Figure 1e). These results clearly showed that the PDCoV HZYH-2019 had been successfully isolated from fecal samples of sows.

### 3.2. Genetic Variation Study of PDCoV Based on Sequence Alignment and ML Phylogenetic Analysis

**Figure 2:**
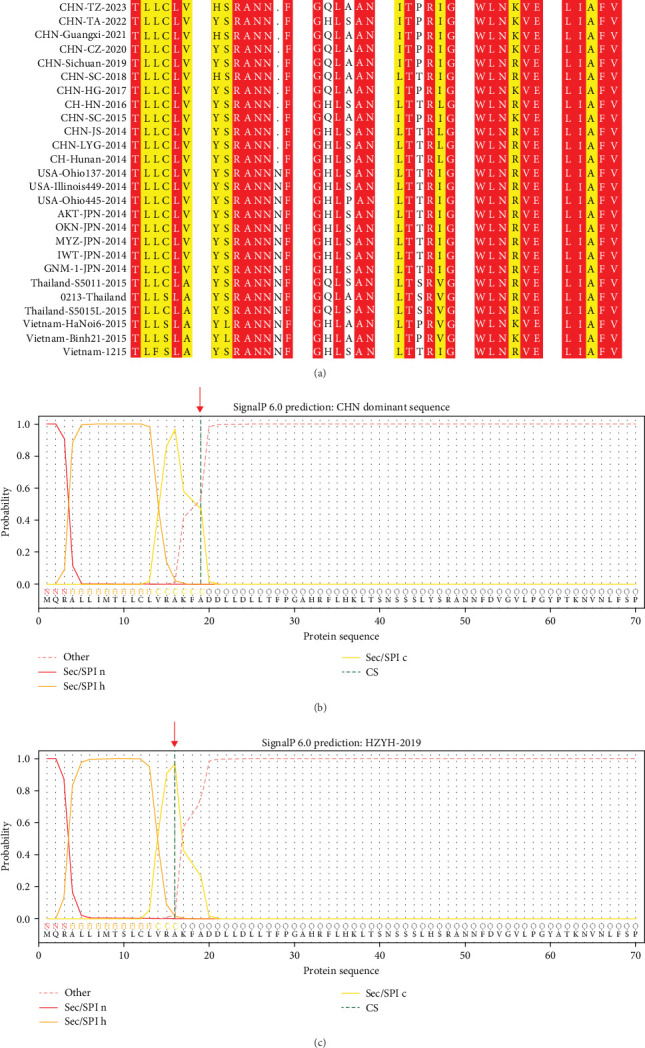
Characterization of the PDCoV HZYH-2019 Spike (S) protein sequence and prediction of its signal peptide. (a) Comparative sequence analysis of the PDCoV HZYH-2019 with other PDCoV S proteins. (b) Signal peptide prediction for the S proteins of most Chinese strains. (c) Signal peptide prediction for the S proteins of PDCoV HZYH-2019.

The genomic sequence of the PDCoV HZYH-2019 strain was compared to 142 PDCoV sequences available in GenBank. The analysis revealed that the nucleotide sequence homology of the full-length genome of PDCoV HZYH-2019 with other PDCoV reference sequences ranged from 96.99% to 98.84%. For the S gene, nucleotide sequence homology ranged from 95.72% to 98.53%, while the amino acid sequence homology ranged from 95.69% to 99.14%. Additionally, PDCoV HZYH-2019 shares a characteristic feature with other Chinese PDCoV strains: a deletion of three nucleotides at positions 148–150 in the S gene, corresponding to the deletion of one asparagine residue in the asparagine trimer at amino acid positions 50–52 (Figure 2a). This codon deletion serves as a genetic marker distinguishing Chinese strains from U.S. strains [40]. In addition, the frequency of the H228Q, S230A, L665I, T667P, and R1089K mutations increased between 2014 and 2023 (Figure 2a). Notably, the S protein of PDCoV HZYH-2019 exhibited two unique mutations at positions L10S and A1010V compared to other strains. Specifically, the mutation at the L10S position results in an earlier cleavage of the signal peptide (Figure 2b,c).

**Figure 3:**
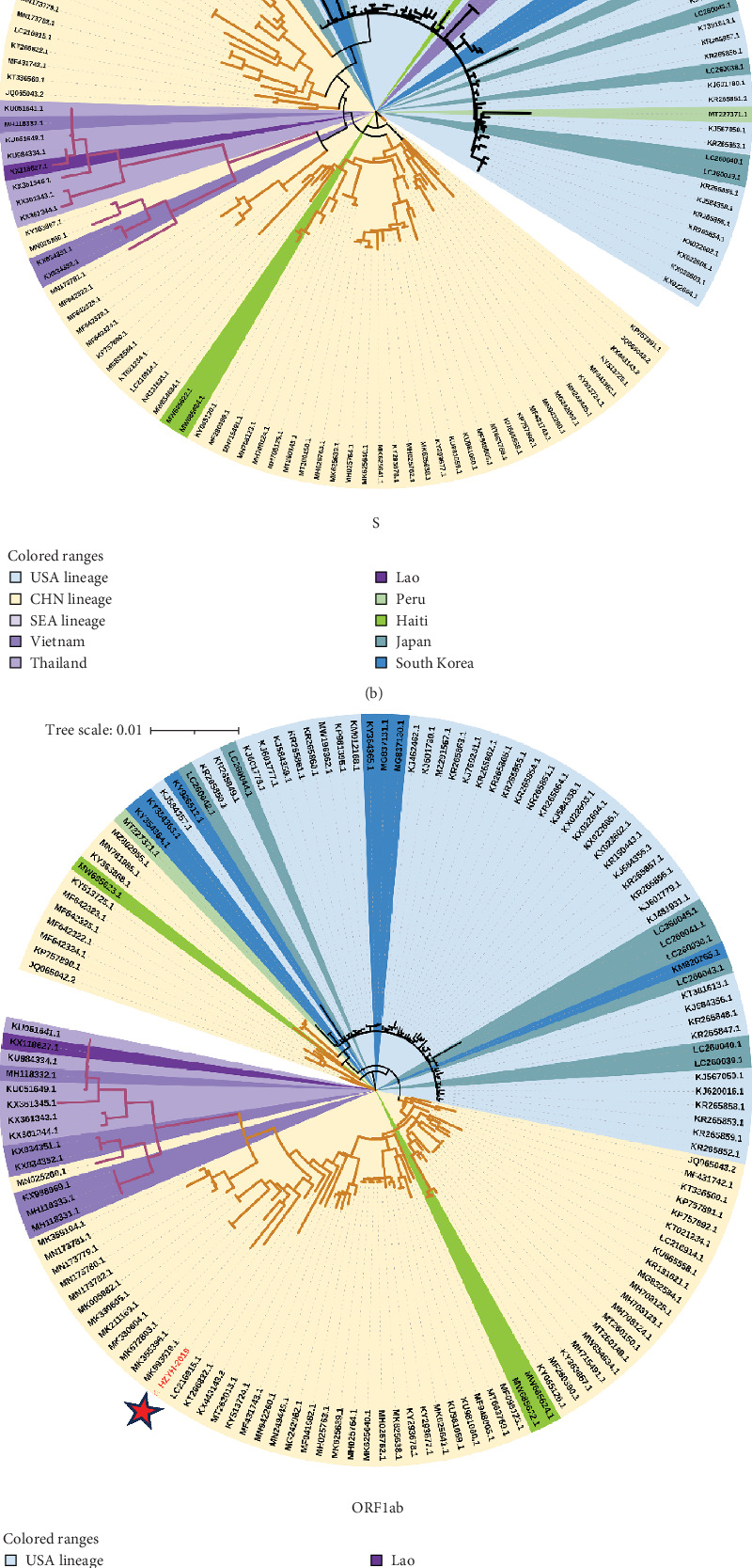
ML phylogenetic analysis of PDCoV HZYH-2019. The phylogenetic tree was constructed based on (a) the full genome, (b) the S gene, (c) the open reading frames 1a and 1b (ORF1ab), and (d) the envelope (E), membrane (M), and nucleocapsid (N) genes.

An ML phylogenetic tree based on the full genome of PDCoV was constructed. PDCoV can be categorized into three main lineages: the Chinese lineage (comprising all Chinese strains), the American lineage (including strains from the United States, Peru, Japan, and South Korea), and the Southeast Asian lineage (comprising strains from Thailand, Vietnam, and Laos). The PDCoV-HZYH-2019 strain identified by our laboratory clustered with Chinese strains and exhibited the highest homology with MF095123 (CHN-HG-2017) at 98.69%. American strains formed a monophyletic cluster. Asian strains were more dispersed, with Korean and Japanese strains clustering within the American lineage (Figure 3a). A Chinese strain MN025260, collected in Southeast Asia Genealogy. Four Chinese strains (MZ802955, MN781985, KY363868, KY513725) were closely related to the American lineage, indicating the presence of American lineage PDCoV strains in China. In addition to strains from these regions, three recently reported Haitian strains isolated from children (MW685622, MW685624, MW685623) were analyzed. Two of these strains were placed in the Chinese lineage, while the third was placed in the American lineage (Figure 3a). Specifically, MW685622 and MW685624 showed genome homology with the Chinese Tianjin strain KY065120 at 99.76% and 99.72%, respectively, whereas MW685623 showed 99.2% genome homology with the American Arkansas strain KR150443.

In addition to the ML phylogenetic tree based on the full genome of PDCoV, separate ML phylogenetic trees were constructed for the S (Figure 3b), ORF1ab (Figure 3c), and E-M-N genes (Figure 3d). The topologies of the ML trees for the full genome, ORF1ab, and E-M-N genes were largely consistent. However, in the ML tree for the S gene, a separation was observed within the Southeast Asian lineage. Specifically, three Vietnamese strains (KX998969, MH118331, and MH118333) appeared as outliers, clustering with the American lineage. This may be due to genetic recombination events.

### 3.3. Evolutionary Rate and Geographic Origin of PDCoV as Inferred From S Gene Analysis

**Figure 4:**
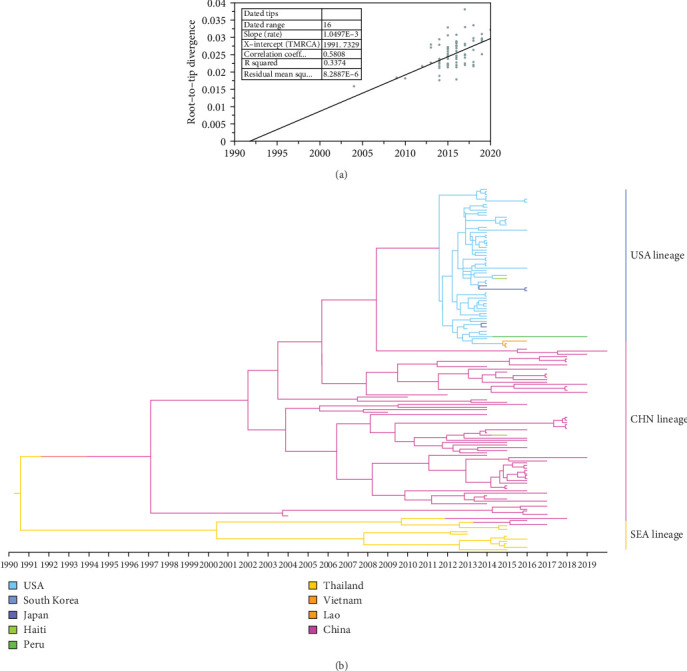
Bayesian differentiation time estimation for PDCoV. (a) Temporal signal testing based on an ML tree of PDCoV S gene. (b) Maximum clade credibility (MCC) tree of PDCoV S gene.

The temporal signal of the PDCoV S gene ML tree was evaluated using TempEst software. The analysis indicated a sufficiently strong temporal signal (correlation coefficient = 0.58, *R*^2^ = 0.34), allowing the use of a molecular clock model to estimate a time-calibrated phylogenetic tree (Figure 4a). Based on path sampling estimation results, an uncorrelated lognormal relaxed clock model and a Bayesian skyline coalescent tree prior model were selected. The MCC tree constructed from the S gene closely resembles the ML tree and can be divided into three major lineages: the Chinese lineage, the American lineage, and the Southeast Asian lineage (Figure 4b). Analysis of the MCC tree suggests that PDCoV likely originated in Asia (either China or Southeast Asia) and subsequently spread from the Chinese lineage to other regions. The estimated time to the most recent common ancestor (tMRCA) of PDCoV is approximately 1990, with a 95% highest posterior density (HPD) interval ranging from 1975 to 2001. The average evolutionary rate of the S gene is estimated to be 1.438 × 10^−3^ nucleotide substitutions per site per year, with a 95% HPD interval of 1.038 × 10^−3^ to 1.849 × 10^−3^.

### 3.4. Recombination and Host-Switching Events Underlying the Avian Origins of PDCoV

**Figure 5:**
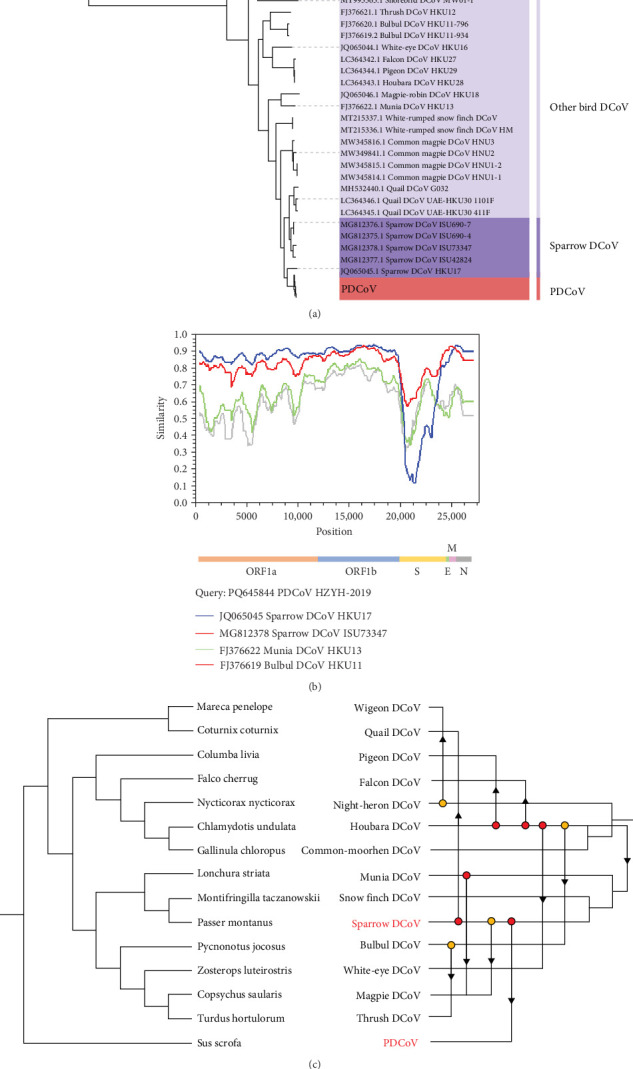
Cross-host transmission of deltacoronaviruses. (a) ML phylogenetic tree of the deltacoronaviruses genome (b). Recombination analysis of PDCoV, with events detected using the Recombination Detection Program (RDP v.4.0.3) with default settings. (c) Analysis of host-virus relationships and reconstruction of cophylogenetic trees using Jane.v.4.

To investigate the genetic diversity of deltacoronaviruses in different hosts, an ML phylogenetic tree was constructed based on the whole genomes of 143 PDCoV and 27 ADCoV strains, using infectious bronchitis virus (IBV) as the outgroup. The results indicate that deltacoronaviruses can be divided into three groups: the PDCoV group, the sparrow deltacoronaviruses (SpDCoV) group, and the other bird deltacoronavirus group, with PDCoV being more closely related to SpDCoV (Figure 5a).

Given the close genomic relationship between PDCoV and SpDCoV, and the fact that other members of the deltacoronavirus genus have been detected in birds, it is hypothesized that ADCoV might be the genetic source of PDCoV. Phylogenetic analysis shows that SpDCoV HKU17 is the closest to PDCoV at the whole-genome level. However, S gene alignment reveals that the amino acid sequence similarity of the S protein between SpDCoV HKU17 and PDCoV is only 58%, whereas SpDCoV ISU73347 shares over 80% similarity with PDCoV. Additionally, bulbul deltacoronavirus (BuDCoV) HKU11 and munia deltacoronavirus (MuDCoV) HKU13 show over 70% similarity with PDCoV. To further explore the origin of PDCoV, recombination analysis was conducted on these closely related ADCoVs. The results suggest that PDCoV may have originated from a recombination event between SpDCoV HKU17 and SpDCoV ISU73347, with the S gene region derived from SpDCoV ISU73347 and the remainder from SpDCoV HKU17 (Figure 5b).

Based on these findings, it is proposed that PDCoV may have undergone cross-species transmission from birds to mammals. Using JANE to analyze the host-virus relationship, five events were identified: cospeciation, duplication, duplication with host switch, loss, and failure to diverge. Each event was assigned a cost value, and the total cost was minimized to assess the congruence between host and virus phylogenies. As shown in Figure 5c, solid dots and arrows represent duplication and host-switching events, indicating frequent host switching of ADCoV among birds. Additionally, host switching between SpDCoV and PDCoV suggests that SpDCoV might have undergone a host switch from sparrows to pigs, leading to the emergence of PDCoV.

### 3.5. Codon Usage Bias and Evolutionary Implications in Deltacoronaviruses

The frequency of synonymous codon usage varies among different species, reflecting codon usage bias, which results from the combined effects of mutation and selection [33]. Quantifying codon bias can help in understanding the evolutionary processes of species [34]. Therefore, a statistical analysis of the codon usage in the CDS of the PDCoV and ADCoV genomes was conducted.

**Figure 6:**
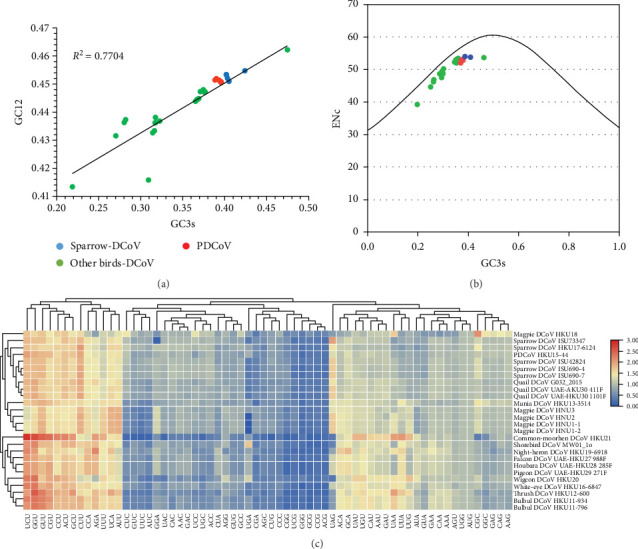
Analysis of codon bias in the coding sequences of deltacoronaviruses. (a) Scatter plot with GC3 content on the *x*-axis and GC12 on the *y*-axis, with *R*^2^ indicating their linear relationship. Different colored dots represent different viruses. (b) Effective number of codons (ENC) plotted against GC3S (frequency of G+C at synonymous third codon positions); ENC values between 20 and 61 indicate varying selection preferences for synonymous codons. Continuous curves show expected values assuming no selection beyond the GC component. (c) Heatmap of relative synonymous codon usage (RSCU), illustrating frequently used codons (red squares) versus less frequently used ones (blue squares).

The calculations indicate that the average GC content of deltacoronaviruses ranges from 0.35 to 0.47, with the average GC12 content ranging from 0.41 to 0.46 and the average GC3 content ranging from 0.22 to 0.47. The variation in GC content is primarily associated with changes in GC3. The neutrality plot of GC3 and GC12 shows a strong correlation with a good fit (*R*^2^ = 0.7704) (Figure 6a), suggesting that PDCoV, SpDCoV, and other avian CoV groups share similar evolutionary characteristics. The ENc values of deltacoronaviruses ranged from 39.3 to 54.1, with a median of 52.4. All observed values were below the standard curve, indicating a relatively weak codon usage bias (Figure 6b). This suggests that, in addition to mutational pressure, other factors, such as selection also influence the codon usage patterns of deltacoronaviruses. The RSCU heatmap reveals that PDCoV clusters with SpDCoV, indicating that their codon usage preferences are notably similar (Figure 6c). This observation further supports the hypothesis that PDCoV may have evolved from ADCoV. In general, the most frequently used codons were those ending in U, such as UCU, GGU, GUU, CGU, CCU, ACU, GCU, and CUU. Conversely, codons ending in G, such as CGG, UCG, GGG, GCG, and CCG, were among the least frequently used (Figure 6c).

### 3.6. Pathogenicity and Clinical Manifestations of PDCoV-HZYH-2019 in Neonatal Piglets

**Figure 7:**
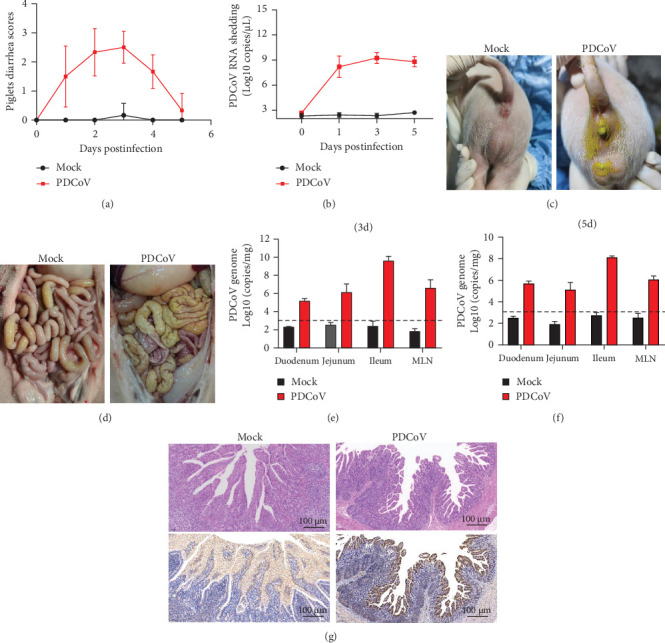
Clinical evaluation and pathological changes in piglets infected with PDCoV HZYH-2019. (a) The scoring system for piglet diarrhea is as follows: 0 = normal, 1 = pasty stool, 2 = semiliquid diarrhea resembling cow dung, 3 = severe watery or liquid diarrhea. (b) RT-qPCR detection of PDCoV fecal shedding in piglets. (c) Diarrhea observed in 5-day-old piglets at 2 dpi with PDCoV or DMEM. (d) Gross pathology assessed at 3 dpi. Viral load of PDCoV infection in the small intestine and mesenteric lymph nodes at 3 dpi (e) and 5 dpi (f). The data are presented as the mean ± standard deviation. (g) Histopathological changes (H&E staining) and PDCoV N protein expression in the ileum of piglets at 3 dpi (magnification: 100x).

To evaluate the pathogenicity of PDCoV HZYH-2019 in pigs, 5-day-old healthy piglets were randomly divided into two groups. One group was orally administered 10^6^ TCID_50_/mL of PDCoV HZYH-2019, while the control group received DMEM. In the infected group, piglets began to exhibit varying degrees of diarrhea from 1 dpi. At 2–3 dpi, half of the piglets developed severe watery diarrhea (Figure 7a,c), but they gradually recovered thereafter. No deaths occurred during the study (Table 1).

Virus shedding in feces and virus distribution in the body were also assessed. PDCoV RNA was detected in the feces of all infected piglets at 1 dpi, peaking at 3 dpi and then declining until 5 dpi (Figure 7b). Three piglets from each group were necropsied on 3 and 5 dpi. Gross examination at 3 dpi showed that the small intestine of the infected group was markedly thin-walled, transparent, and gaseous with a yellow, watery content (Figure 7d). No other lesions were observed in the organs of the control group or in other organs of the infected group.

Viral RNA was analyzed in the duodenum, jejunum, ileum, and mesenteric lymph nodes, with the highest levels detected in the ileum, which decreased by 5 dpi (Figure 7e,f). Histopathological examination using HE staining showed villous atrophy and fragmentation in the ileum of the infected group. Consistent with these findings, IHC revealed abundant PDCoV N protein in the cytoplasm of intestinal villous cells. No histopathological changes or PDCoV N antigen were observed in the negative control group (Figure 7g).

## 4. Discussion

The isolation and characterization of the PDCoV strain HZYH-2019 from a severe diarrhea outbreak in piglets provide crucial insights into the virus's biological behavior and its impact on swine health. This study sheds light on the growth kinetics, genomic characteristics, and pathogenicity of the virus, offering a deeper understanding of its impact.

Firstly, our findings on the growth kinetics of PDCoV HZYH-2019 demonstrated strong cell line specificity, with enhanced replication in LLC-PK1 cells compared to ST cells. This observation suggests that differences in cell surface receptors or intracellular factors may play a significant role in viral replication efficiency. Furthermore, genomic analysis revealed high nucleotide sequence similarity with other PDCoV strains, but also some genetic variation, notably in the S protein, which is critical for host cell invasion. A trinucleotide deletion in the S gene, shared by HZYH-2019 and other Chinese strains, distinguishes these strains from US strains [40], although it does not appear to affect the virus's structure or antigenicity. Interestingly, a specific mutation in the *S* protein's signal peptide region did not significantly impact viral replication or infectivity, suggesting compensatory mechanisms may maintain its function. Further experiments are certainly needed to demonstrate the potential effects of the mutation.

In terms of phylogenetic relationships, our analyses classified PDCoV into three lineages: China, the United States, and Southeast Asia. Four Chinese strains were clustered in the American lineage, likely due to trade in pork and related products between the US and China, which facilitates cross-border transmission of these strains. Additionally, the detection of PDCoV in Haitian children has raised concerns, which might be linked to historical pig imports from the US and pork trade with China [41]. MCC tree analysis based on the S gene suggested that PDCoV originated in Asia around 1990 and spread from China to the United States. Although outbreaks of porcine diarrhoeal disease caused by PDCoV first occurred in the United States, a retrospective study found that it was present in diarrhoeal pigs in Anhui Province as early as 2004 [42].

To date, all known members of the genus Deltacoronavirus, except PDCoV, have been found in birds, indicating that birds may be the natural hosts. Our study suggests that PDCoV likely originated from recombination events among different SpDCoVs and cross-species transmission from sparrows to pigs. Given that sparrows and other birds often share environments with domesticated mammals, sparrows could serve as an intermediate host for PDCoV, with the true natural hosts being other wild bird species. Sparrows are frequently present on pig farms, especially in colder months, and their droppings or contaminated feed could facilitate the transmission of deltacoronavirus to pigs.

Previous studies have established that PDCoV strains vary in clinical outcomes, with certain highly pathogenic strains causing acute watery diarrhea, dehydration, and mortality in neonatal piglets, thereby posing substantial economic losses in swine production. In our study, neonatal piglets infected with PDCoV HZYH-2019 developed diarrhea at 1 dpi, peaked in severity at 2–3 dpi, and recovered completely without mortality. This phenotype is consistent with other low pathogenicity Chinese strains, such as CZ2020, CHN-HN-2014, and CHN-HG2017 [43–45]. While HZYH-2019 induces significant intestinal lesions with viral replication confined to the small intestine and mesenteric lymph nodes, its self-limiting course contrasts sharply with the fatal outcomes reported for highly pathogenic strains like ZD 2022 from Zhejiang province and CHN-ANHZ-2024 from Anhui province [46, 47]. This phenotypic divergence underscores the critical influence of strain-specific genetic signatures on pathogenicity. First, the L10S mutation in the HZYH-2019S protein shifts the predicted signal peptide cleavage site (Figure 2b,c), potentially altering S protein processing and receptor-binding domain (RBD) accessibility during viral entry. Second, genomic analysis revealed multiple non-synonymous mutations in the S1 subunit of CHN-ANHZ-2024, a region critical for host receptor binding [46]. Mutations in the S1 subunit may influence receptor binding efficiency or alter host tropism [48]. To evaluate the functional impact of these mutations on receptor usage, further studies utilizing pseudotyped viruses or competitive binding assays are required. More importantly, the hypervirulent ZD 2022 strain harbors two additional N-glycosylation sites (N526 and N1008) in its S protein compared to most other PDCoVs, which may facilitate immune evasion by shielding neutralizing epitopes while improving host cell attachment flexibility [47, 49]. Understanding these mechanisms could inform targeted attenuation strategies for vaccine development and enhance molecular surveillance for emergent hypervirulent variants.

## 5. Conclusions

This study provides critical insights into the PDCoV strain HZYH-2019, emphasizing its potential for cross-species transmission and its pathogenic effects in piglets. The successful isolation and characterization of the virus, along with its demonstrated growth advantage in specific cell lines, underscore the importance of understanding its biology and evolution. The genomic analysis, revealing high homology with other Chinese strains and evidence of recombination events with avian coronaviruses, suggests a complex evolutionary history that may facilitate host-switching. These findings are crucial for informing future strategies aimed at preventing and controlling PDCoV outbreaks, ultimately contributing to the development of effective vaccines and biosecurity measures to protect the swine industry and mitigate zoonotic risks.

## Figures and Tables

**Table 1:** Clinical observation records of 5-day-old pigs challenged with PDCoV strain HZYH-2019.

DPI	Clinical observation	Fecal consistency			
		Normal	Pasty stool	Semiliquid diarrhea	Severe diarrhea
0	All active and eating well	6/6	0/6	0/6	0/6
1	50% lethargy and anorexia	1/6	2/6	2/6	1/6
2	All lethargy, 83% anorexia	0/6	1/6	2/6	3/6
3	All lethargy, anorexia	0/6	0/6	3/6	3/6
4^a^	All active and eating well	0/3	1/3	2/3	0/3
5	All active and eating well	2/3	1/3	0/3	0/3

^a^Three pigs were necropsied at 3 days postinoculation.

## Data Availability

The data used to support the findings of this study are available within the paper. The PDCoV HZYH-2019 genome sequence was submitted to GenBank under accession number PQ645844.

## Supporting Information

Supporting Information Table S1. PDCoV sequence information used in the phylogenetic analyses. Table S2. ADCoV sequence information used in the phylogenetic analysis.
