# Supplementary material for: Genetic and Pathogenic Characterization of a Porcine Deltacoronavirus Strain Isolated in Zhejiang Province, China
Source: Transbound Emerg Dis. 2025 May 18;2025:4084814. doi: 10.1155/tbed/4084814 (PMC12103969; doi:10.1155/tbed/4084814)
Supplement: Supporting Information — Table S1. PDCoV sequence information used in the phylogenetic analyses. Table S2. ADCoV sequence information used in the phylogenetic analysis. [file 4084814.f1.docx]

**Table S1. PDCoV sequence information**

| GenBank number | Strain | Region |
| --- | --- | --- |
| KT336560 | CHN-HN-2014 | China |
| JQ065042 | HKU15-44 | China |
| JQ065043 | HKU15-155 | China |
| KJ462462 | OH1987 | USA |
| KJ481931 | PDCoV/USA/Illinois121/2014 | USA |
| KJ567050 | USA/IA/2014/8734 | USA |
| KJ584355 | IL2768 | USA |
| KJ584356 | SD3424 | USA |
| KJ584357 | KY4813 | USA |
| KJ584358 | PA3148 | USA |
| KJ584359 | NE3579 | USA |
| KJ601777 | PDCoV/USA/Illinois133/2014 | USA |
| KJ601778 | PDCoV/USA/Illinois134/2014 | USA |
| KJ601779 | PDCoV/USA/Illinois136/2014 | USA |
| KJ601780 | PDCoV/USA/Ohio137/2014 | USA |
| KJ620016 | MI6148 | USA |
| KJ769231 | SdCV/USA/OhioCVM1/2014 | USA |
| KM012168 | Michigan/8977/2014 | USA |
| KM820765 | KNU14-04 | Korea |
| KP757890 | CHN-AH-2004 | China |
| KP757891 | CHN-HB-2014 | China |
| KP757892 | CHN-JS-2014 | China |
| KP981395 | USA/IL/2014/026PDV | USA |
| KR131621 | PDCoV/CHJXNI2/2015 | China |
| KR150443 | USA/Arkansas61/2015 | USA |
| KR265847 | USA/Minnesota442/2014 | USA |
| KR265848 | USA/Minnesota214/2014 | USA |
| KR265849 | USA/Michigan447/2014 | USA |
| KR265850 | USA/Michigan448/2014 | USA |
| KR265851 | USA/Indiana453/2014 | USA |
| KR265852 | USA/Illinois449/2014 | USA |
| KR265853 | USA/Minnesota/2013 | USA |
| KR265854 | USA/Minnesota454/2014 | USA |
| KR265855 | USA/Minnesota455/2014 | USA |
| KR265856 | USA/Illinois272/2014 | USA |
| KR265857 | USA/Illinois273/2014 | USA |
| KR265858 | USA/NorthCarolina452/2014 | USA |
| KR265859 | USA/Minnesota159/2014 | USA |
| KR265860 | USA/Nebraska209/2014 | USA |
| KR265861 | USA/Nebraska210/2014 | USA |
| KR265862  KR265863  KR265864  KR265865  KT381613  MW685622  MW685623  MW685624 | USA/Ohio444/2014  USA/Ohio445/2014  USA/Minnesota292/2014  USA/Iowa459/2014  OH11846  PDCoV/Haiti/Human/0081-4/2014  PDCoV/Haiti/Human/0256-1/2015  PDCoV/Haiti/Human/0329-4/2015 | USA  USA  USA  USA  USA  Haiti  Haiti  Haiti |

(Continue Table S1)

| GenBank number | Strain | Region | | |
| --- | --- | --- | --- | --- |
| MW196362 | USA/IL/2014/026PDV | | USA |  |
| MZ291567 | OH-FD22 | | USA |  |
| MW854634 | 104-553 | | China |  |
| KT021234 | CH/SXD1/2015 | | China |  |
| KT266822 | CH/Sichuan/S27/2012 | | China |  |
| MZ802955 | CH-HLJ-20 | | China |  |
| KU051641 | PDCoV/Swine/Thailand/S5011/2015 | | Tailand |  |
| KU051649  KU984334 | PDCoV/Swine/Thailand/S5015L/2015  TT_1115 | | Tailand Tailand |  |
| KX118627 | PDCoV/2016/Lao | | Laos |  |
| KX361343 | PDCoV/0213/Thailand | | Laos |  |
| KX361344 | PDCoV/0213/Thailand | | Laos |  |
| KX361345 | PDCoV/2015/Thailand | | Laos |  |
| KX443143 | CH-01 | | China |  |
| KX834351 | PDCoV/Swine/Vietnam/HaNoi6/2015 | | Vietnam |  |
| KX834352 | PDCoV/Swine/Vietnam/Binh21/2015 | | Vietnam |  |
| KX998969 | VN_1215 | | Vietnam |  |
| KY065120 | CHN/Tianjin/2016 | | China |  |
| KY293677 | CH/JXJGS01/2016 | | China |  |
| KY293678 | CH/JXJGS02/2016 | | China |  |
| KY354363 | DH1 | | Korea |  |
| KY354364 | DH2 | | Korea |  |
| KY363867 | CHN-GD16-03 | | China |  |
| KY363868 | CHN-GD16-05 | | China |  |
| KY364365 | KNU16-07 | | Korea |  |
| KY513724 | CH/Hunan/2014 | | China |  |
| KY513725 | CH/Jiangsu/2014 | | China |  |
| KY926512 | KNU16-11 | | Korea |  |
| LC216914 | S579N | | China |  |
| LC216915 | S582N | | China |  |
| LC260038 | AKT/JPN/2014 | | Japan |  |
| LC260039 | GNM-1/JPN/2014 | | Japan |  |
| LC260040 | GNM-2/JPN/2014 | | Japan |  |
| LC260041 | IWT/JPN/2014 | | Japan |  |
| LC260042 | MYZ/JPN/2014 | | Japan |  |
| LC260043 | OKN/JPN/2014 | | Japan |  |
| LC260044 | YMG/JPN/2014 | | Japan |  |
| LC260045 | HKD/JPN/2016 | | Japan |  |
| MF041982 | SHJS/SL/2016 | | China |  |
| MF095123 | CHN-HG-2017 | | China |  |
| MF280390 | CHN-GD-2016 | | China |  |
| MF431742  MT260150  MT263013  MT663769  MT260149  MN942260 | GD  HNZK-04-P15  CHN-HN-17  CHN-TS1-2019  HNZK-04-P5  HeN | | China  China  China  China  China  China |  |

(Continue Table S1)

| GenBank number | Strain | Region |
| --- | --- | --- |
| MF431743 | SD | China |
| MF642322 | CHN/GS/2016/1 | China |
| MF642323 | CHN/GS/2016/2 | China |
| MF642324 | CHN/GS/2017/1 | China |
| MF642325 | CHN/QH/2017/1 | China |
| MF948005 | HB-BD | China |
| MG242062 | CHN-HeB1-2017 | China |
| MG832584 | CHN-HN-1601 | China |
| MG837130 | KNU16-07-P5 | Korea |
| MG837131 | KNU16-07-P10 | Korea |
| MH025762 | CH/JXJGS01/P7 | China |
| MH025763 | CH/JXJGS01/P20 | China |
| MH025764 | CH/JXJGS01/P50 | China |
| MH118331 | VN_0116 | Vietnam |
| MH118332 | VN_0416 | Vietnam |
| MH118333 | VN_1215 | Vietnam |
| MH708123 | HNZK-02 | China |
| MH708124 | HNZK-04 | China |
| MH708125 | HNZK-06 | China |
| MH715491 | PDCoV/CHGD/2016 | China |
| MK005882 | Swine/CHN/SC/2018/1 | China |
| MK211169 | CHN/Sichuan/2017 | China |
| MK330604 | CHN/Sichuan/2017 | China |
| MK330605 | CHN/Sichuan/2018 | China |
| MK355396 | CHN-SC2015 | China |
| MK359104 | CHN-GX01-2018 | China |
| MK572803 | SCNC201705 | China |
| MK625638 | CH/JXJGS01/2016 | China |
| MK625639 | CH/JXJGS01/2016 | China |
| MK625640 | CH/JXJGS01/2016 | China |
| MK625641 | CH/JXJGS01/2016 | China |
| MK993519 | CHN/Sichuan/2019 | China |
| MN025260 | CH/GX/1468B/2017 | China |
| MN173779 | CHN-GX11-2018 | China |
| MN173780 | CHN-GX12-2018 | China |
| MN173781 | CHN-GX81-2018 | China |
| MN173782 | CHN-GX09-2018 | China |
| MN249445 | CHN-JS-2017 | China |
| MN781985 | CHzmd2019 | China |
| MT227371 | PDCoV/Peru/isolate/2019 | Peru |
|  |  |  |

**Table S2. ADCoV sequence information**

| GenBank  number | Strain | Host | Region |
| --- | --- | --- | --- |
| FJ376619 | Bulbul CoV HKU11-934 | Pycnonotus jocosus | China |
| FJ376620 | Bulbul CoV HKU11-796 | Pycnonotus jocosus | China |
| FJ376621 | Thrush CoV HKU12 | Turdus hortulorum | China |
| FJ376622 | Munia CoV HKU13 | Lonchura striata | China |
| JQ065044 | White-eye CoV HKU16 | Zosterops luteirostris | China |
| JQ065045 | Sparrow CoV HKU17 | Passer montanus | China |
| JQ065046 | Magpie-robin CoV HKU18 | Copsychus saularis | China |
| JQ065047 | Night-heron CoV HKU19 | Nycticorax nycticorax | China |
| JQ065048 | Wigeon CoV HKU20 | Mareca penelope | China |
| JQ065049 | Common-moorhen CoV HKU21 | Gallinula chloropus | China |
| LC364342 | Falcon CoV HKU27 | Falco cherrug | UAE |
| LC364343 | Houbara CoV HKU28 | Chlamydotis undulata | UAE |
| LC364344 | Pigeon CoV HKU29 | Columba livia | UAE |
| LC364345 | Quail CoV HKU30 411F | Coturnix coturnix | UAE |
| LC364346 | Quail CoV HKU30 1101F | Coturnix coturnix | UAE |
| MG812375 | Sparrow CoV ISU690-4 | Passer montanus | USA |
| MG812376 | Sparrow CoV ISU690-7 | Passer montanus | USA |
| MG812377 | Sparrow CoV ISU42824 | Passer montanus | USA |
| MG812378 | Sparrow CoV ISU73347 | Passer montanus | USA |
| MH532440 | Quail CoV G032 | Coturnix coturnix | Poland |
| MT215336 | White-rumped snow finch CoV | Montifringilla taczanowskii | China |
| MT215337 | White-rumped snow finch CoV HM | Montifringilla taczanowskii | China |
| MT993565 | Shorebird CoV MW01_1 | Arenaria interpres | Austalian |
| MW345814 | Magpie CoV HNU1-1 | Copsychus saularis | China |
| MW345815 | Magpie CoV HNU1-2 | Copsychus saularis | China |
| MW345816 | Magpie CoV HNU3 | Copsychus saularis | China |
| MW349841 | Magpie CoV HNU2 | Copsychus saularis | China |
